# The consumption of chili peppers and the risk of colorectal cancer: a matched case-control study

**DOI:** 10.1186/s12957-019-1615-7

**Published:** 2019-04-17

**Authors:** Yalan Yang, Jing Zhang, Noel S. Weiss, Linwen Guo, Li Zhang, Yanqi Jiang, Yanfang Yang

**Affiliations:** 1 Department of Epidemiology and Biostatistics, West China School of Public Health and West China Fourth Hospital, Sichuan University, Chengdu, Sichuan China; 2 Department of Environmental Health and Occupational Medicine, West China School of Public Health and West China Fourth Hospital, Sichuan University, Chengdu, Sichuan China; 3 Department of Epidemiology, School of Public Health, University of Washington, Seattle, USA

**Keywords:** Colorectal cancer, Chili peppers, Diets, Case-control study

## Abstract

**Background:**

Chili peppers have properties that plausibly could either increase or decrease a person’s risk of developing colorectal cancer, but their consumption in relation to disease risk has not been well studied. We sought to explore the association between chili peppers intake and the risk of colorectal cancer.

**Methods:**

Eight hundred subjects (400 cases with colorectal cancer and 400 controls) were enrolled in this study. Cases were primarily colorectal cancer patients diagnosed by histopathology at the Department of Intestinal Surgery, Sichuan Cancer Hospital from July 2010 to May 2012. Controls were people receiving routine medical examinations from the Zhonghe Community Health Service Center during the same period of time. An in-person interview was used to collect demographic characteristics, lifestyle, and dietary habits of the subjects in reference to the 10 years prior to disease diagnosis. Conditional logistic regression was conducted to examine the possible association between the risk of colorectal cancer and chili peppers consumption.

**Results:**

Compared with persons who consumed chili peppers ≤ 2 times per week, those who consumed chili peppers 3–7 times per week (OR = 1.2, 95% CI 0.75–2.0, *P* = 0.413) and > 7 times per week (OR = 1.4, 95% CI 0.84–2.2, *P* = 0.205) were not at an increased risk of colorectal cancer.

**Conclusions:**

The results suggest that the consumption of chili peppers does not increase or decrease the risk of colorectal cancer. This question warrants being re-addressed in a study in which there is prospective ascertainment of dietary characteristics.

## Background

Colorectal cancer(CRC)is at present the third most common malignancy in the world [1]. Historically, the incidence rates have been different in various countries. With changes in lifestyle and the westernization of dietary habits, the incidence of and mortality from CRC in China have increased in recent years. In 2015, there were 376,000 new cases and 191,000 deaths in China [2].

The chili pepper has been a part of the human diet for thousands of years [3]. It was often used as a main condiment in Sichuan province. The chili pepper contains capsaicinoids, derivatives of phenylpropanoid compounds, which are responsible for their intense hot taste and pungency, with E-capsaicin (8-methyl-N-vanillyl-trans-6-nonenamide) being the major capsaicinoid present in chili peppers [4]. A study has been observed that capsaicin can downregulate the expression of COX-2 and B-catenin mRNA, promoting apoptosis through caspase 3 activation and inhibiting the proliferation of cells [5, 6]. However, intake of chili peppers also could act as an irritant, leading to an inflammatory reaction that could favor the development of malignancy. While consumption of chili peppers has been observed to be associated with a reduction in incidence of all forms of cancer combined [7], this question has not been examined specifically for CRC.

## Objects and methods

### Study participants

We made the matching principle before participants recruiting. Controls were matched 1:1 to cases by gender and age (± 3 years). Eight hundred subjects (400 cases with CRC and 400 controls) were enrolled who were (1) Han Chinese and (2) had lived in the province of Sichuan for more than 10 years. We excluded persons with (1) a history of other cancers; (2) a history of digestive system diseases; and (3) several other conditions (i.e., hypertension, diabetes, hyperlipidemia). Cases were primarily CRC patients diagnosed by histopathology at the Department of Intestinal Surgery, Sichuan Cancer Hospital (Chengdu, Sichuan, China) from July 2010 to May 2012. The age of cases was no limit. Controls were people from the Zhonghe Community Health Service Center (Chengdu, Sichuan, China) seen during the same period of time who had no earlier history of cancer. We obtained written informed consent from all subjects. The study protocol was approved by the West China Fourth Hospital of Sichuan University (West China School of Public Health).

### Survey

A face-to-face interview was conducted with subjects. If the patient could not answer the questions, family members were asked to serve as proxy respondents. The interview sought information on demographic characteristics, family history of CRC, and dietary habits and lifestyle characteristics during the 10 years before diagnosis.

Specifically, we asked about consumption of (1) chili peppers; (2) red meat (e.g., beef, lamb, and pork). According to the Dietary Guideline and Balance Diet Pagoda for Chinese Residents, the recommended daily consumption for red meat is 50–75 g [8]. This study defined the intake amount of > 50 g as one intake of red meat [9, 10] , > 7 times per week being defined as “high” consumption [9]; (3) cured meat (with more than 30 g, ≥ 24 times per year being defined as “high” consumption [7]); (4) pickles (≥ 3 times per week being defined as “high” consumption [11]); (5) bean products(≥ 3 times per week being defined as “high” consumption [11]); (6) high-fat foods (e.g., animal liver, kidney, brain, large intestine, frequency per month, > 2 times per month being defined as “high” consumption); (7) sweetmeats (e.g., dessert, candy, ice cream, chocolate, frequency per week, ≥ 3 times per week being defined as “high” consumption [11]); (8) fruit (frequency per week, ≥ 3 times per week being defined as “high” consumption [11]); and (9) vegetable (frequency per week, > 7 times per week being defined as “high” consumption [11]).

We also asked study participants about smoking regularly (smoking more than one cigarette per day for at least 6 months [12]), drinking alcohol regularly (drinking one time per day or more and drinking for a month or more [13]), tea-drinking regularly (drinking tea one time per day or more and drinking for a month or more [14]), and exercise regularly (weekly exercise ≥ 3 times for ≥ 20 min [11]).

### Statistical analysis

EpiData 3.1 software and SPSS 22.0 software were used for statistical analysis. Referring to the classification of dietary factors in other studies [9], Chili peppers were classified according to four-digit intervals (the upper quartile and the lower quartile) of per week consumption. Other diets and lifestyle were all classified referring to the previous researches of our group [9, 11]. The odds ratios (OR) with 95% confidence intervals (CI) for chili pepper in CRC risk were calculated using conditional logistic regression (LR) and adjusted by other diets and lifestyle. Some factors were determined as the adjustments based on their *P* value (*P < 0.05*). Other factors were identified as the adjustments because they were mostly thought to be related to CRC in other studies. The significance level was *P* < 0.05.

## Results

### Characteristics of participants

For the cases and controls, 233 (58.2%) were males and 167 (41.8%) were females. The mean ages were 55.73 ± 11.08 years old in the cases and 55.74 ± 11.19 years old in the controls. There were no significant differences in education, profession, and household per capita annual income between cases and controls (*P* > 0.05). Demographic characteristics of cases and controls are presented in Table 1.

**Table 1:** Distribution of demographic characteristics between cases and controls

Variables	Cases *N*(%)	Controls *N*(%)	*χ* ^*2*^	*P*
Education				
Primary school and below	151(37.75)	175(43.75)	5.576	0.062
Junior-senior high school and secondary school	216(54.00)	183(45.75)		
Junior college and above	33 (8.25)	42(10.50)		
Profession				
Enterprises and institutions	104(26.00)	111(27.75)	1.287	0.864
Manufacturing industry	78(19.50)	85(21.25)		
Transport industry	12 (3.00)	13 (3.25)		
Agriculture, forestry, fishery, and animal husbandry	145(36.25)	131(32.75)		
Others	61(15.25)	60(15.00)		
Household per capita annual income(yuan per year)				
< 10000	134(33.50)	118(29.50)	1.530	0.465
10000–20000	159(39.75)	166(41.50)		
> 20000	107(26.75)	116(29.00)		

### Diets and lifestyle

In Table 2, we present the distribution of consumption of all diets and lifestyle in cases and controls: chili peppers, red meat, cured meat, pickles, tea, beans, fruits, vegetable, and sweetmeats, as well as their distributions with respect to lifestyle and a family history of CRC. There were significant differences in the distribution of chili peppers, red meat, cured meat, pickles, tea-drinking regularly, beans, fruit, sweetmeats, daily sitting time, exercise regularly, and family history of CRC between the cases and controls (*P* < 0.05).

**Table 2:** Distribution of dietary and lifestyle characteristics of cases and controls

Variables	Cases, *N*(%)	Controls, *N*(%)	*χ* ^*2*^	*P*
Chili peppers (times per week)				
≤ 2	95(23.75)	136(34.00)	17.601^a^	< 0.001
3–7	114(28.50)	130(32.50)		
> 7	191(47.75)	134(33.50)		
Red meat(times per week)				
≤ 7	144(36.00)	214(53.50)	24.773	< 0.001
> 7	256(64.00)	186(46.50)		
Cured meat(times per year)				
< 24	144(36.00)	240(60.00)	46.154	< 0.001
≥ 24	256(64.00)	160(40.00)		
Pickles(times per week)				
< 3	151(37.75)	259(64.75)	58.356	< 0.001
≥ 3	249(62.25)	141(35.25)		
Tea-drinking regularly				
No	249(62.25)	211(52.75)	7.386	0.007
Yes	151(37.75)	189(47.25)		
Beans(times per week)				
< 3	318(79.50)	271(67.75)	14.220	< 0.001
≥ 3	82(20.50)	129(32.25)		
Fruits(times per week)				
< 3	186(46.50)	150(37.50)	6.650	0.010
≥ 3	214(53.50)	250(62.50)		
High-fat food(times per week)				
≤ 2	273(68.25)	282(70.50)	0.477	0.490
> 2	127(31.75)	118(29.50)		
Sweetmeats(times per week)				
< 3	296(74.00)	323(80.75)	5.205	0.023
≥ 3	104(26.00)	77(19.25)		
Vegetables(times per week)				
≤ 7	56(14.00)	61(15.25)	0.250	0.617
> 7	344(86.00)	339(84.75)		
Daily sitting time(hour)				
< 8	296(74.00)	337(84.25)	12.721	< 0.001
≥ 8	104(26.00)	63(15.75)		
Smoking regularly				
No	226(56.50)	230(57.50)	0.082	0.775
Yes	174(43.50)	170(42.50)		
Drinking regularly				
No	273(68.25)	274(68.50)	0.006	0.939
Yes	127(31.75)	126(31.50)		
Exercise regularly				
No	247(61.75)	151(37.75)	46.081	< 0.001
Yes	153(38.25)	249(62.25)		
Family history of CRC				
No	365(91.25)	391(97.75)	16.258	< 0.001
Yes	35 (8.75)	9 (2.25)		

Table 3 presents the results of a multivariate analysis of chili peppers in relation to CRC in which we adjusted for consumption of red meat, cured meat, pickles, tea, beans, fruit, vegetables, high-fat foods, sweetmeats; and for daily sitting time, smoking regularly, drinking regularly, exercise regularly, and family history of CRC. There were significant differences among red meat, cured meat, pickles, tea, bean, daily sitting time, exercise regularly, and family history of CRC (*P* < 0.05). However, there were no significant differences among chili peppers, fruit, vegetables, high-fat food, sweetmeats, smoking regularly, and drinking regularly (*P* > 0.05). We observed that weekly consumption of chili peppers 3 to 7 times (OR = 1.2 95% *CI* 0.75–2.0) and > 7 times (OR = 1.4 95 %*CI* 0.84–2.2) were not associated with the risk of CRC. (Table 3)

**Table 3:** ORs and 95%CIs of chili peppers for CRC

Variable	Cases *N*(%)	Controls *N*(%)	OR^a^(95% CI)	*P*
Chili peppers(times per week)				
≤ 2	95(23.75)	136(34.00)	Reference	
3–7	114(28.50)	130(32.50)	1.2(0.75–2.0)	0.413
> 7	191(47.75)	134(33.50)	1.4(0.84–2.2)	0.205

^a^Adjusted for intake of red meat, cured meat, pickles, tea, bean, fruit, vegetables, high-fat food, and sweetmeats; for daily sitting time, smoking regularly, drinking regularly, and exercise regularly; and for family history of CRC

Table 4 presents the ORs associated with intake of chili peppers in different subgroups defined by intake of red meat, cured meat, high-fat foods; daily sitting time; smoking regularly, and drinking regularly. Across these subgroups, generally, there was no association between the consumption of chili peppers and the risk of CRC(*P* > 0.05). There was one possible exception; among persons with a relatively low consumption of cured meat, we observed an increased risk associated with weekly consumption of chili peppers 3–7 times (OR = 1.2 95% CI 0.78–2.0) and > 7 times (OR = 1.6 95% CI 1.1–2.5), whereas no such increases were seen among persons with a relatively high consumption of cured meat.

**Table 4:** ORs and 95% CIs of chili pepper for CRC in different groups

Subgroups	Variable	Cases *N*(%)	Controls *N*(%)	OR(95% CI)	*P*
Low red meat (≤ 7 times per week)	Chili peppers (times per week)				
	≤ 2	39(27.08)	88(41.12)	Reference	
	3–7	55(38.19)	80(37.38)	1.0(0.69–1.6)	0.826
	> 7	50(34.72)	46(21.50)	1.3(0.80–2.0)	0.326
High red meat (> 7 times per week)	Chili peppers (times per week)				
	≤ 2	56(21.88)	48(25.81)	Reference	
	3–7	59(23.05)	50(26.88)	0.96(0.65–1.4)	0.835
	> 7	141(55.08)	88(47.31)	1.1(0.76–1.5)	0.736
Low cured meat (< 24 times per year)	Chili peppers (times per week)				
	≤ 2	36(25.00)	101(42.08)	Reference	
	3–7	38(26.39)	78(32.50)	1.2(0.78–2.0)	0.362
	> 7	70(48.61)	61(25.42)	1.6(1.1–2.5)	0.022
High cured meat (≥ 24 times per year)	Chili peppers (times per week)				
	≤ 2	59(23.05)	35(21.88)	Reference	
	3–7	76(29.69)	52(32.50)	0.90(0.63–1.3)	0.539
	> 7	121(47.27)	73(45.63)	0.89(0.65–1.2)	0.499
Low high-fat food (≤ 2 times per week)	Chili peppers (times per week)				
	≤2	75(27.47)	104(36.88)	Reference	
	3-7	69(25.27)	87(30.85)	0.98(0.70–1.4)	0.907
	>7	129(47.25)	91(32.27)	1.1(0.84–1.5)	0.404
High high-fat food (> 2 times per week)	Chili peppers (times per week)				
	≤ 2	20(15.75)	32(27.12)	Reference	
	3–7	45(35.43)	43(36.44)	1.1(0.65–1.9)	0.700
	> 7	62(48.82)	43(36.44)	1.1(0.66–1.9)	0.666
Low daily sitting (< 8 h)	Chili peppers (times per week)				
	≤ 2	70(23.65)	118(35.01)	Reference	
	3–7	91(30.74)	113(33.53)	1.1(0.78–1.5)	0.624
	> 7	135(45.61)	106(31.45)	1.2(0.92–1.7)	0.157
High daily sitting (≥ 8 h)	Chili peppers (times per week)				
	≤ 2	25(24.04)	18(28.57)	Reference	
	3–7	23(22.12)	17(26.98)	0.98(0.53–1.8)	0.944
	> 7	56(53.85)	28(44.44)	0.95(0.57–1.6)	0.852
Not regular drinking	Chili peppers (times per week)				
	≤2	69(25.27)	105(38.32)	Reference	
	3–7	84(30.77)	95(34.67)	1.1(0.76–1.5)	0.761
	>7	120(43.96)	74(27.01)	1.2(0.88–1.6)	0.262
Regular drinking	Chili peppers (times per week)				
	≤ 2	26(20.47)	31(24.60)	Reference	
	3–7	30(23.62)	35(27.78)	0.93(0.54–1.6)	0.776
	> 7	71(55.91)	60(47.62)	1.0(0.64–1.6)	0.922
Not regular smoking	Chili peppers (times per week)				
	≤2	57(25.22)	88(38.26)	Reference	
	3-7	68(30.09)	77(33.48)	1.1(0.78–1.6)	0.525
	>7	101(44.69)	65(28.26)	1.2(0.85–1.7)	0.298
Regular smoking	Chili peppers (times per week)				
	≤ 2	38(21.84)	48(28.24)	Reference	
	3–7	46(26.44)	53(31.18)	0.89(0.57–1.4)	0.606
	> 7	90(51.72)	69(40.59)	1.1(0.71–1.6)	0.792

## Discussion

Capsaicin concentration is related to the variety and maturity of chili peppers and its concentration can change. E-capsaicin is the major capsaicin in chili peppers. A study shows a greater than 25-fold variation in E-capsaicin levels among individual peppers, ranging from about 0.04 μg/mL to 1 μg/mL [15]. In general, the concentration of capsaicin ranges from 0.1 to 1% in chili peppers.

The results of some studies suggested that intake of capsaicin may predispose to one or more forms of gastrointestinal cancer. Dietary administration of capsaicin produced duodenal tumors in Swiss albino mice [16]. An epidemiological study conducted in Mexico observed that consumers of chili peppers were at higher risk for gastric cancer than non-consumers [17]. In addition, excessive consumption of chili peppers may irritate colonic mucosa. Ingestion of large amounts of capsaicin has been reported to cause histopathological and biochemical changes, including erosion of gastric mucosa and hepatic necrosis [18].

However, the results of other studies suggested that intake of capsaicin might reduce cancer risk. Studies in the laboratory had observed capsaicin to interfere with the action of some chemical carcinogens, such as aflatoxin B_1_ and the tobacco-specific nitrosamine, 4-(methylnitrosamino)-1-(3-pyridyl)-1-butanone [19], and also to inhibit the proliferation of CRC cells and induce apoptosis and necrosis of cancer cells.

Initial work by Hoch-Ligeti showed that rats fed diets containing 10% chili pepper developed liver tumors [20]. The validity of this study, however, was questioned because of possible contamination of the experimental diet with the potent hepatocarcinogen aflatoxin B_1_. Results of Surh and Lee’s preliminary study indicated that repeated application of capsaicin on the shaved backs of female ICR mice following an initial dose of 7,12-dimethylbenz[a]anthracene led to no significant enhancement of papilloma formation in the skin, compared with the control animals that received the carcinogen alone. Summarizing the relevant studies, Surh and Lee suggested that although a minute amount of capsaicin displays few or no deleterious effects, heavy ingestion of the compound has been associated with necrosis, ulceration, and even carcinogenesis [18]. According to previous studies, capsaicin has both carcinogenic and anticancer effects.

In our study, we did not observe a significant difference in chili peppers: compared to persons consuming chili peppers no more than twice each week, the relative risk in those whose weekly intake was 3–7 times and > 7 times was 1.2 (95% CI 0.75–2.0) and 1.4 (95% CI 0.84–2.2), respectively. In fact, the relationship between chili peppers and CRC was indeed complex. The chili pepper is a spicy food, and excess consumption may damage to colonic mucosa. The long-term damage is related to the occurrence of CRC. In addition, capsaicin may play the weak carcinogenesis role in causing CRC. In another context, chili peppers are beneficial. Capsaicin also has an anticancer effect. Besides, ingestion of chili peppers can promote digestive juice to secrete and accelerate bowel movements [21], which may reduce the risk of CRC.

Generally, there was little difference in the size of the OR associated with consumption of chili peppers across different subgroups, though we did observe an increased risk associated with weekly consumption of chili peppers 3–7 times (OR = 1.2 95% CI 0.78–2.0) and > 7 times (OR = 1.6 95% CI 1.1–2.5) among persons with a relatively low consumption of cured meat. With our existing knowledge, we considered it suggested a link or an interaction between chili pepper and cured meat. Lots of capsaicin may weakly suppress the chemical carcinogens, such as PAH found in cured meat. It needs further study to elucidate the mechanism associated with this observation.

Interpretation of the results must take into account some limitations of our study. First, family members responding to proxy respondents about diet habits over the past 10 years is a limitation. And the study collected dietary information only for the past 10 years, which may be less relevant to risk than intake earlier in life. Second, even for intake during the past 10 years, there can be non-differential misclassification of exposure status when interviews are used to obtain the information on frequency of intake. Third, the study measured only the times of chili peppers intake, not the quantity consumed. Finally, the referent category used in our analysis included some persons who did consume chili peppers, just at a lower frequency than 3 times per week. However, an analysis in which the referent category has no chili pepper intake could not be done, due to the fact that among the study participants, there was virtually no one who did not consume chili peppers.

## Conclusions

In summary, the results suggest that the consumption of chili peppers does not increase or decrease the risk of CRC. The results of studies with prospective and more detailed ascertainment of diet, in populations (such as this one) in which a high intake of chili peppers is present, are needed to clarify.

## Abbreviations

CRC: Colorectal cancer
